# Impact of sex differences in co‐morbidities and medication adherence on outcome in 25 776 heart failure patients

**DOI:** 10.1002/ehf2.13113

**Published:** 2020-11-28

**Authors:** Muhammed T. Gürgöze, Onno P. van der Galiën, Marlou A.M. Limpens, Stefan Roest, René C. Hoekstra, Arne S. IJpma, Jasper J. Brugts, Olivier C. Manintveld, Eric Boersma

**Affiliations:** ^1^ Department of Cardiology Thorax Center, Erasmus MC, University Medical Center Rotterdam Rotterdam The Netherlands; ^2^ Zilveren Kruis Achmea Leusden The Netherlands; ^3^ Department of Epidemiology Erasmus MC, University Medical Center Rotterdam Rotterdam The Netherlands; ^4^ Department of Pathology Erasmus MC, University Medical Center Rotterdam Rotterdam The Netherlands

**Keywords:** Heart failure, Co‐morbidity, Medication adherence, Hospitalisation, Mortality, Big data

## Abstract

**Aims:**

Health insurance claims (HIC) databases in the Netherlands capture unselected patient populations, which makes them suitable for epidemiological research on sex differences. Based on a HIC database, we aimed to reveal sex differences in heart failure (HF) outcomes, with particular focus on co‐morbidities and medication.

**Methods and results:**

The Achmea HIC database included 14 517 men and 11 259 (45%) women with a diagnosis treatment code for chronic HF by January 2015. We related their sex, co‐morbidities, and medication adherence (medication possession rate >0.8) with the primary endpoint (PE) of all‐cause mortality or HF admission during a median follow‐up of 3.3 years, using Cox regression. Median age of men and women was 72 and 76 years, respectively. Prevalence of co‐morbidities and use of disease‐modifying drugs was higher in men; however, medication adherence was similar. At the end of follow‐up, 35.1% men and 31.8% women had reached the PE. The adjusted hazard ratio for men was 1.25 (95% confidence interval: 1.19–1.30). A broad range of co‐morbidities was associated with the PE. Overall, these associations were stronger in women than in men, particularly for renal insufficiency, chronic obstructive pulmonary disease/asthma, and diabetes. Non‐adherence to disease‐modifying drugs was related with a higher incidence of the PE, with similar effects between sexes.

**Conclusions:**

In a representative sample of the Dutch population, as captured in a HIC database, men with chronic HF had a 25% higher incidence of death or HF admission than women. The impact of co‐morbidities on the outcome was sex dependent, while medication adherence was not.

## Introduction

Randomized controlled trials (RCTs) are broadly accepted as the golden standard to evaluate the efficacy and safety of pharmacological treatment. However, RCTs usually have strict inclusion and exclusion criteria, which makes their representativeness for clinical practice questionable. For example, in the cardiovascular domain, including heart failure (HF), RCT participants are selected from a predominantly (younger) male patient population,^1^ whereas (elderly) women and those with more complex diseases are often underrepresented.^2^ Hence, RCTs insufficiently cover the heterogeneity of the HF population, including the broad variety of socio‐economic factors, the presence of (multiple) co‐morbidities, and medication adherence among men and women. Consequently, clinical trial databases are generally less suitable for studying any sex‐specific effect of these factors on HF outcomes.

In the Netherlands, over 99% of the population has (basic) health insurance^3^ as such insurance is mandatory by law. Thus, health insurance claims (HIC) databases in the Netherlands capture truly representative (random) samples of patient populations. The sample sizes are large, and data on co‐morbidities and medication claims are collected in a systematic way.^4^ We used the HIC database of Zilveren Kruis Achmea, one of several health insurance companies, to describe the key characteristics of an unselected population of men and women with chronic heart failure (CHF) and to study the sex‐specific impact of co‐morbidities and medication on prognosis.

## Methods

### Study design and patient selection

A retrospective, observational study was carried out using anonymous HIC data of Zilveren Kruis Achmea, the largest insurance company in the Netherlands comprising about 5.1 million people (30%) of the Dutch population.^5^ The database contained data from January 2012 to April 2018. The period until December 2014 was used for patient selection and determining characteristics of the study sample. Between January 2015 and April 2018, the outcomes of the selected patients were determined.

**Figure 1:**
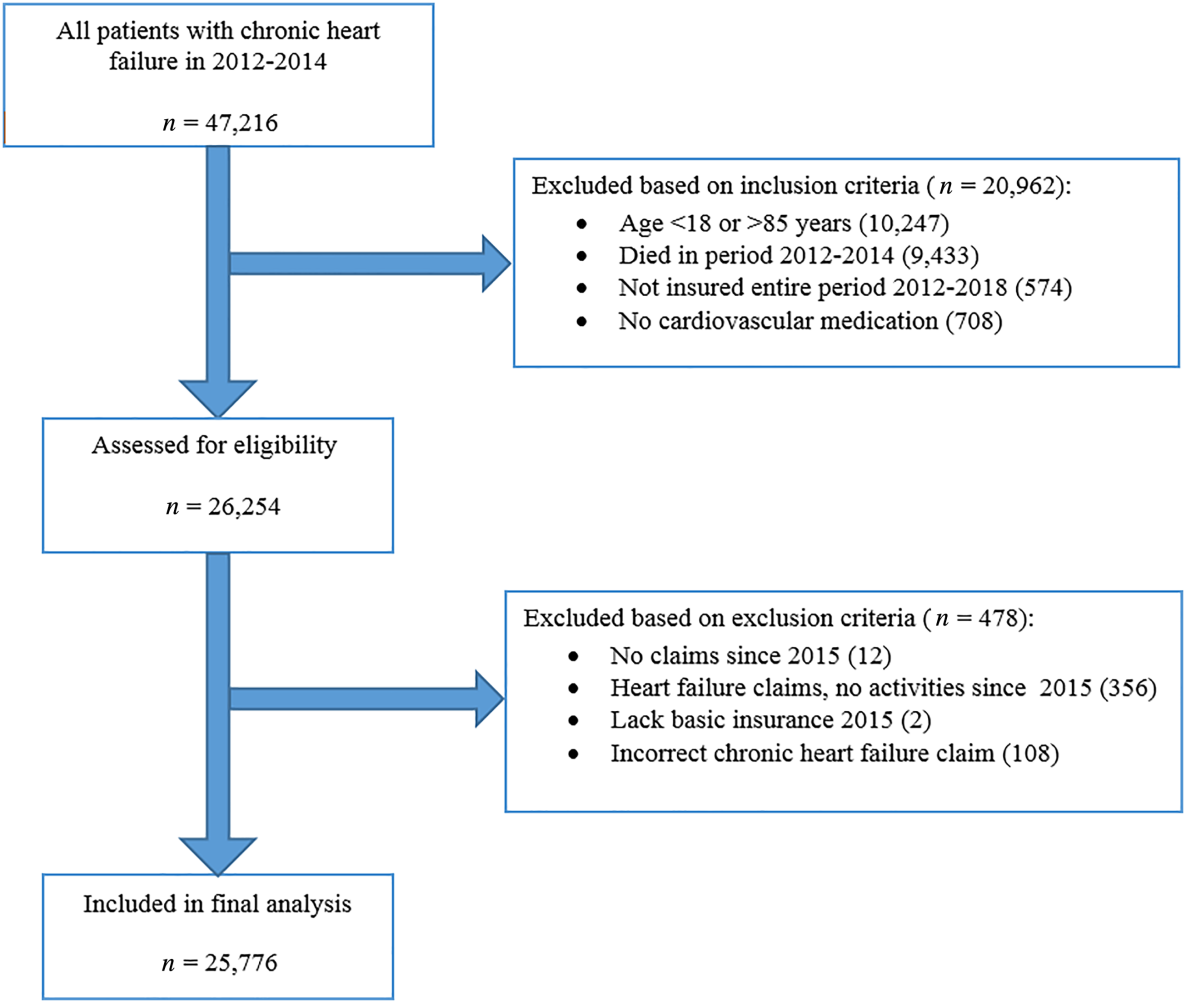
Flowchart of study population selection.

We identified 25 776 patients aged 18–85 years with a diagnosis treatment code for CHF and still alive by January 2015 (*Figure* *1*). These patients had a CHF‐related claim according to the national diagnosis treatment classification system called ‘Diagnose Behandeling Combinatie’ (DBC), which is a combination of the International Classification of Diseases, 10th revision (ICD‐10)^6^ and treatment applied. Additionally, they had used at least one prescription drug within the cardiovascular system (‘C’) based on World Health Organization Anatomical Therapeutic Chemical Classification index and Defined Daily Dose (WHO ATC/DDD) in the same period.^7^

According to the European Society of Cardiology Heart Failure guidelines,^8^ CHF patients should visit their treating physician at least once per year. To improve data quality, we therefore excluded patients who lacked any HF insurance claims after January 2015. Patients who switched to another insurance company between 2012 and 2018 were also excluded.

### Co‐morbidity selection

Co‐morbidities were identified using a combination of the adapted diagnosis‐related group (DRG) classification and the pharmacy‐based cost group (FKG classification). In the Netherlands, DRG is an ICD‐10‐based system, used to determine health care costs in relation to specific diseases. The FKG is a classification method for medication type/dose in relation to chronic diseases, which is used in the national risk equalization model. It is used to adjust capitation payments by the health care insurer to the health care provider.

### Medication use and adherence

Medication use and adherence was determined for the period 2012–2014. Extensive pharmacy data were available. The indication for prescribing a certain drug of selection is not known. Medication adherence was determined using the medication possession ratio (MPR).^9^ This ratio was defined as the amount of pills corrected for different dosage schemes, the prescribed daily dose supplied, divided by the time (days) between two supply dates. Patients may switch drugs within the same class. Therefore, to obtain more reliable MPR estimates, medications were grouped into ATC classes. Consecutively, MPR was averaged over the total supply period per ATC group and categorized based on a threshold of 0.80, above which a patient was considered adherent to prescribed medication.^9^

### Outcomes and follow‐up

The primary endpoint (PE) was a composite of all‐cause mortality and hospitalisation for HF, based on the DBC system. Secondary endpoints were the two components of this composite. Biological sex differences in co‐morbidities and medication adherence in relation to the PE were of particular interest. Mortality was retrieved from the civil registry. HF admissions were not adjudicated by a committee.

For additional information on patient selection, definition of socio‐demographic factors, co‐morbidities, medication adherence, and outcomes, see Supporting Information, *Appendix S1*.

### Statistical analysis

Continuous variables are presented as median and interquartile range, and sex differences were evaluated using Mann–Whitney tests. Categorical variables are presented as counts and percentages, and sex differences were evaluated using *χ* ^2^‐tests.

The associations between sex, co‐morbidities, and medication adherence and the PE were assessed using Cox proportional hazard regression models, with adjustment for other predefined, clinically relevant baseline variables, including age, marital status, socio‐economic status, income level, and time since last CHF‐related outpatient/clinic visit. The proportional hazard assumption was satisfied for each variable. We investigated potential effect modification by sex via an interaction term in the Cox models.

For all tests, a *P*‐value < 0.05 was considered statistically significant. Data were analysed using R Statistical Software Version 3.4.2 (Vienna, Austria) and the "survival" package.

## Results

### Baseline characteristics and co‐morbidities

The analysis set included 14 517 (55%) men and 11 259 (45%) women. Women were significantly older (76 vs. 72 years). Baseline characteristics are described in *Table* *1*. More than a third of the patients (35%) had three or more co‐morbidities. Arrhythmia (41%), ischaemic heart disease (33%), diabetes mellitus type 1 or 2 (DM1/2) (25%), malignancy (25%), and chronic obstructive pulmonary disease (COPD)/asthma (17%) were most common. Men had a higher prevalence of these top five co‐morbidities. Women had a higher prevalence of valvular heart disease, hypertensive disease, thyroid dysfunction, and depression. Men and women had a similar prevalence of renal insufficiency (RI) and cerebrovascular disease.

**Table 1:** Baseline characteristics

Characteristics	All patients *N* = 25 776	Men *N* = 14 517	Women *N* = 11 259	*P*‐value
Age (years), median (IQR)	74 (66–80)	72 (65–79)	76 (67–81)	**<0.001**
Sex, *n* (%)				
Men	14 517 (56)			
Women	11 259 (44)			
Marital status, *n* (%)				**<0.001**
Married	8697 (34)	6007 (41)	2690 (24)	
Unknown	8428 (33)	4616 (32)	3812 (34)	
Widow/widower	3802 (15)	1211 (8)	2591 (23)	
Never married	3040 (12)	1823 (13)	1217 (11)	
Divorced	1809 (7)	860 (6)	949 (8)	
SES score, median (IQR)	−0.37 (−1.17 to 0.47)	−0.15 (−1.17 to −0.35)	−0.21 (−1.26 to −0.40)	**<0.001**
Income level, median (IQR)	5.0 (2.0–7.0)	5.0 (2.0–7.0)	5.0 (2.0–7.0)	**<0.001**
Duration since last visit^a^, *n* (%)				**<0.001**
0–6 months	2993 (12)	1617 (11)	1376 (12)	
6–12 months	3327 (13)	1822 (13)	1505 (13)	
1–2 years	6975 (27)	3827 (26)	3148 (28)	
>2 years	12 481 (48)	7251 (50)	5230 (46)	
Co‐morbidities^b^, *n* (%)				**<0.001**
0	2614 (10)	1363 (9)	1251 (11)	
1	6552 (25)	3562 (25)	2990 (27)	
2	7604 (30)	4419 (30)	3185 (28)	
≥3	9006 (35)	5173 (35)	3833 (34)	
History of co‐morbidities^b^, *n* (%)				
Arrhythmia	10 569 (41)	6409 (44)	4160 (37)	**<0.001**
Ischaemic heart disease	8445 (33)	5045 (35)	3400 (30)	**<0.001**
Diabetes mellitus 1/2	6500 (25)	3789 (26)	2711 (24)	**<0.001**
Malignancy	6328 (25)	3703 (26)	2625 (23)	**<0.001**
COPD/asthma	4433 (17)	2609 (18)	1824 (16)	**<0.001**
Hypercholesterolaemia	3753 (15)	2410 (17)	1343 (12)	**<0.001**
Valve disease	3607 (14)	1864 (13)	1743 (15)	**<0.001**
Renal insufficiency	3110 (12)	1757 (12)	1353 (12)	0.833
Hypertensive disease	2128 (8)	1030 (7)	1098 (10)	**<0.001**
Cerebrovascular disease	1941 (8)	1100 (8)	841 (7)	0.745
Thyroid dysfunction	1792 (7)	595 (4)	1197 (11)	**<0.001**
Depression	1420 (6)	570 (4)	850 (8)	**<0.001**

COPD, chronic obstructive pulmonary disease; IQR, interquartile range; SES, socio‐economic status.

Significant *P*‐values are in bold.

a Duration since last chronic heart failure outpatient clinic visit or admission in period 2012–2014.

b Only pre‐selected clinically relevant co‐morbidities.

### Medication use and adherence

Baseline medication use is shown in *Table* *2*. The vast majority of patients used angiotensin‐converting enzyme‐inhibitors/angiotensin receptor blockers (ACE‐I/ARBs) (85%), beta‐blockers (83%), or diuretics (83%), in particular loop diuretics (71%) and mineralocorticoid receptor antagonists (MRAs) (40%). Up to a third used calcium blockers (33%), and digoxin was prescribed in 19% of patients. Almost a quarter of patients (23%) used oral nitrates (isosorbide). Cardiovascular medication use, in particular disease‐modifying drugs, was somewhat higher in men, except for calcium blockers, diuretics, and digoxin.

**Table 2:** Medication use

	All patients *N* = 25 776	Men *N* = 14 517	Women *N* = 11 259	*P*‐value
Medication^a^, *n* (%)				
ACE‐inhibitors/ARBs	21 846 (85)	12 623 (87)	9223 (82)	**<0.001**
Beta‐blockers	21 397 (83)	12 267 (85)	9130 (81)	**<0.001**
Calcium blockers	8446 (33)	4522 (31)	3924 (35)	**<0.001**
Diuretics	21 292 (83)	11 654 (80)	9638 (86)	**<0.001**
Loop diuretics	18 286 (71)	10 056 (69)	8230 (73)	**<0.001**
Thiazide diuretics	6685 (26)	3220 (22)	3465 (31)	**<0.001**
Mineralocorticoid receptor antagonists	10 434 (40)	5956 (41)	4478 (40)	**0.042**
Digoxin	4980 (19)	2723 (19)	2257 (20)	**0.009**
Amiodarone	2304 (9)	1524 (10)	780 (7)	**<0.001**
Doxazosin	963 (4)	513 (4)	450 (4)	0.052
Ivabradine	636 (2)	389 (3)	247 (2)	**0.013**
Hydralazine	119 (0)	83 (1)	36 (0)	**0.003**
Nitrates (isosorbide)	5946 (23)	3493 (24)	2453 (22)	**<0.001**
Anti‐coagulants	14 482 (56)	8493 (59)	5989 (53)	**<0.001**
Anti‐platelets	13 422 (52)	8094 (56)	5328 (47)	**<0.001**
Lipid‐lowering therapy	17 221 (67)	10 452 (72)	6769 (60)	**<0.001**
Glucose‐lowering therapy	7828 (30)	4498 (31)	3330 (30)	**0.015**

ACE, angiotensin‐converting enzyme; ARBs, angiotensin receptor blockers.

Significant *P*‐values are in bold.

a Medication use in period 2012–2014.

Baseline medication adherence based on MPR is presented in *Table* *3*. The adherence was largely similar in men and women in each ATC group. A (borderline) statistically significant difference between both sexes was observed for ACE‐I/ARBs, beta‐blockers, loop diuretics, and MRAs.

**Table 3:** Medication adherence

ATC group	Adherence	All patients^a^	Men^a^	Women^a^	*P*‐value
ACE‐I/ARBs, *n* (%)		*n* = 21 846	*n* = 12 623	*n* = 9223	
	Adherent	18 048 (82.6)	10 482 (83.0)	7566 (82.0)	**0.010**
	Non‐adherent	1327 (6.1)	782 (6.2)	545 (5.9)	
	Unknown	2471 (11.3)	1359 (10.8)	1112 (12.1)	
Beta‐blockers, *n* (%)		*n* = 21 397	*n* = 12 267	*n* = 9130	
	Adherent	17 737 (82.9)	10 158 (82.8)	7579 (83.0)	**0.033**
	Non‐adherent	1214 (5.7)	736 (6.0)	478 (5.2)	
	Unknown	2446 (11.4)	1373 (11.2)	1073 (11.8)	
Calcium blockers, *n* (%)		*n* = 8446	*n* = 4522	*n* = 4924	
	Adherent	5782 (68.5)	3127 (69.2)	2655 (67.7)	0.258
	Non‐adherent	507 (6.0)	273 (6.0)	234 (6.0)	
	Unknown	2157 (25.5)	1122 (24.8)	1035 (26.4)	
Diuretics, *n* (%)		*n* = 21 292	*n* = 11 654	*n* = 9638	
	Adherent	15 489 (72.7)	8519 (73.1)	6970 (72.3)	0.403
	Non‐adherent	1858 (8.7)	996 (8.5)	862 (8.9)	
	Unknown	3945 (18.5)	2139 (18.4)	1806 (18.7)	
Loop diuretics, *n* (%)		*n* = 18 286	*n* = 10 056	*n* = 8230	
	Adherent	11 966 (65.4)	6653 (66.2)	5313 (64.6)	**0.039**
	Non‐adherent	2200 (12.0)	1207 (12.0)	993 (12.1)	
	Unknown	4120 (22.5)	2196 (21.8)	1924 (23.4)	
Thiazide diuretics, *n* (%)		*n* = 6685	*n* = 3220	*n* = 3465	
	Adherent	3823 (57.2)	1837 (57.0)	1986 (57.3)	0.751
	Non‐adherent	403 (6.0)	188 (5.8)	215 (6.2)	
	Unknown	2459 (36.8)	1195 (37.1)	1264 (36.5)	
MRA, *n* (%)		*n* = 10 434	*n* = 5956	*n* = 4478	
	Adherent	7357 (70.5)	4242 (71.2)	3115 (69.6)	0.051
	Non‐adherent	903 (8.7)	483 (8.1)	420 (9.4)	
	Unknown	2174 (20.8)	1231 (20.7)	943 (21.1)	
Amiodarone, *n* (%)		*n* = 2304	*n* = 1524	*n* = 780	
	Adherent	1427 (61.9)	963 (63.2)	464 (59.5)	0.197
	Non‐adherent	159 (6.9)	99 (6.5)	60 (7.7)	
	Unknown	718 (31.2)	462 (30.3)	256 (32.8)	
Digoxin, *n* (%)		*n* = 4980	*n* = 2723	*n* = 2257	
	Adherent	3602 (72.3)	1957 (71.9)	1645 (72.9)	0.636
	Non‐adherent	199 (4.0)	114 (4.2)	85 (3.8)	
	Unknown	1179 (23.7)	652 (23.9)	527 (23.3)	
Nitrates (isosorbide), *n* (%)	Refill rate	*n* = 5946	*n* = 3493	*n* = 2453	
	Adherent	2977 (50.1)	1712 (49.0)	1265 (51.6)	0.139
	Non‐adherent	252 (4.2)	148 (4.2)	104 (4.2)	
	Unknown	2717 (45.7)	1633 (46.8)	1084 (45.7)	

ACE‐I, angiotensin‐converting enzyme inhibitor; ARBs, angiotensin receptor blockers; ATC, anatomical therapeutic chemical classification; MRA, mineralocorticoid receptor antagonist.

Significant *P*‐values are in bold.

a Only patients who used medication within the specific ATC group and in period between 2012 and 2014.

### Outcomes

Median follow‐up time was 3.3 years (interquartile range 2.2–3.3). The PE of all‐cause mortality or HF hospitalisation was reached in 8669 patients (34%) (*Table* *4*). A total of 7152 patients (28%) died, and 3179 (12%) were hospitalized. Men had a higher incidence of the PE than women (35.1% vs. 31.8%). Adjusted hazard ratio (aHR) for the composite endpoint was 1.25 (95% confidence interval: 1.19–1.30, *P* < 0.001) for men. Furthermore, men had a higher overall mortality (29% vs. 26%, *P* < 0.001). Although men had higher mortality rates overall, no significant difference in mortality was found between men and women who had been admitted (54% vs. 51%, *P* = 0.101). Men were hospitalized more often for HF (60% of total admission count) compared with women (*P* < 0.001). Sixty‐six per cent had only one HF‐related admission during follow‐up. Average length of stay was 8.1 days for both men and women.

**Table 4:** Heart failure outcomes

HF outcomes	All patients *N* = 25 776	Men *N* = 14 517	Women *N* = 11 259	*P*‐value
Mortality or HF hospitalisation^a^, *n* (%)	8669 (33.6)	5094 (35.1)	3575 (31.8)	**<0.001**
All‐cause mortality, *n* (%)	7152 (27.7)	4222 (29.1)	2930 (26.0)	**<0.001**
HF hospitalisation, *n* (%)	3179 (12.3)	1875 (12.9)	1304 (11.6)	**0.001**
	HF hospitalized *n* = 3179	Men *n* = 1875	Women *n* = 1304	
All‐cause mortality, *n* (%)	1662 (52.3)	1003 (53.5)	659 (50.5)	0.101
HF hospital admissions^b^, *n* (%)	5291	3166	2125	**<0.001**
1 HF‐related admission	2083 (65.5)	1217 (64.9)	866 (66.4)	
2 HF‐related admissions	624 (19.6)	373 (19.9)	251 (19.3)	
≥3 HF‐related admissions	472 (14.8)	285 (15.2)	187 (14.3)	

HF, heart failure.

Significant *P*‐values are in bold.

a Composite endpoint.

b Cumulative frequency of admissions.

### Determinants of the primary endpoint

The patient's age and a broad range of co‐morbidities seemed to be predictive of the PE (*Table* *5*). RI (aHR 1.49), COPD/asthma (aHR 1.46), DM1/2 (aHR 1.33), cerebrovascular disease (aHR 1.29), and malignancy (aHR 1.25) were the most significant determinants of increased risk. Interestingly, hypercholesterolaemia, hypertensive disease, and ischaemic heart disease were associated with a reduced risk. Non‐adherence to disease‐modifying drugs was significantly associated with increased risk, in particular ACE‐I/ARB (aHR 1.17) and MRA (aHR 1.20), but not beta‐blockers.

**Table 5:** Determinants of all‐cause mortality or heart failure admission

		Sex‐specific estimates			
Characteristics	All patients	Men	Women		
	HR (95% CI)	HR (95% CI)	HR (95% CI)		Interaction *P*‐value
Age, years	1.04 (1.04–1.05)^‡^	1.04 (1.04–1.05)^‡^	1.04 (1.04–1.05)^‡^		0.473
Sex, men	1.25 (1.19–1.30)^‡^	—	—		—
Marital status					
Married	Reference	Reference	Reference		
Unknown	0.83 (0.79–0.88)^‡^	0.80 (0.75–0.86)^‡^	0.85 (0.78–0.94)^‡^		0.315
Widow/widower	0.95 (0.89–1.01)	0.87 (0.79–0.96)^†^	0.96 (0.87–1.06)		0.153
Never married	1.20 (1.12–1.30)^‡^	1.41 (1.29–1.54)^‡^	0.90 (0.79–1.03)		**<0.001**
Divorced	1.22 (1.12–1.33)^‡^	1.44 (1.28–1.61)^‡^	1.02 (0.89–1.16)		**<0.001**
Duration since last visit^a^	0.95 (0.92–0.97)^‡^	0.96 (0.93–0.99)*	0.93 (0.89–0.96)^‡^		0.135
Socio‐economic status	0.99 (0.97–1.01)	1.01 (0.99–1.04)	0.96 (0.94–0.99)^†^		**0.005**
Income level (0–10)	0.97 (0.97–0.98)^‡^	0.98 (0.97–0.99)^‡^	0.97 (0.95–0.98)^‡^		0.091
*Co‐morbidities*					
Arrhythmia	0.96 (0.92–1.00)	0.95 (0.89–1.00)^‡^	0.98 (0.92–1.05)^‡^		0.400
Cerebrovascular disease	1.29 (1.20–1.39)^‡^	1.27 (1.16–1.39)^‡^	1.33 (1.19–1.48)^‡^		0.564
COPD/asthma	1.46 (1.39–1.54)^‡^	1.39 (1.31–1.48)^‡^	1.58 (1.47–1.71)		**0.011**
Depression	1.09 (0.99–1.19)	1.05 (0.92–1.21)	1.11 (0.98–1.25)^‡^		0.587
Diabetes mellitus 1/2	1.33 (1.27–1.40)^‡^	1.26 (1.19–1.34)^‡^	1.44 (1.34–1.55)^‡^		**0.004**
Hypercholesterolaemia	0.78 (0.72–0.85)^‡^	0.78 (0.71–0.87)^‡^	0.77 (0.67–0.88)^‡^		0.834
Hypertensive disease	0.85 (0.78–0.92)^‡^	0.83 (0.74–0.94)^†^	0.86 (0.77–0.97)*		0.695
Ischaemic heart disease	0.90 (0.86–0.95)^‡^	0.87 (0.81–0.92)^‡^	0.96 (0.90–1.04)		**0.022**
Malignancy	1.25 (1.19–1.31)^‡^	1.22 (1.15–1.30)^‡^	1.29 (1.19–1.38)^‡^		0.277
Renal insufficiency	1.49 (1.41–1.58)^‡^	1.41 (1.31–1.52)^‡^	1.61 (1.48–1.75)^‡^		**0.018**
Thyroid dysfunction	1.01 (0.93–1.09)	0.91 (0.79–1.03)	1.07 (0.97–1.19)		**0.046**
Valve disease	1.05 (0.99–1.11)	0.98 (0.90–1.06)	1.14 (1.05–1.25)^†^		**0.008**
*Medication use and adherence*					
ACE‐inhibitor/ARB					
Adherent	Reference	Reference	Reference		
Non‐adherent	1.17 (1.06–1.29)^†^	1.20 (1.06–1.35)^†^	1.13 (0.97–1.31)		0.537
Unknown	1.18 (1.10–1.27)^‡^	1.21 (1.10–1.32)^‡^	1.14 (1.03–1.27)*		0.426
Never used	1.11 (1.05–1.19)^‡^	1.16 (1.07–1.26)^‡^	1.06 (0.97–1.16)		0.144
Beta‐blockers					
Adherent	Reference	Reference	Reference		
Non‐adherent	1.09 (0.98–1.20)	1.17 (1.03–1.33)*	0.97 (0.82–1.14)		0.064
Unknown	0.99 (0.92–1.07)	1.02 (0.93–1.12)	0.94 (0.84–1.06)		0.282
Never used	1.05 (0.99–1.12)	1.07 (0.99–1.16)	1.03 (0.94–1.13)		0.531
Calcium blockers					
Adherent	Reference	Reference	Reference		
Non‐adherent	0.98 (0.84–1.14)	1.02 (0.83–1.26)	0.92 (0.73–1.16)		0.502
Unknown	1.04 (0.96–1.13)	1.11 (0.99–1.24)	0.96 (0.85–1.08)		0.076
Never used	0.99 (0.94–1.04)	1.06 (0.99–1.14)	0.90 (0.83–0.97)^‡^		**0.001**
Loop diuretics					
Adherent	Reference	Reference	Reference		
Non‐adherent	1.00 (0.93–1.08)	1.04 (0.94–1.14)	0.95 (0.85–1.06)		0.232
Unknown	1.03 (0.97–1.09)	0.99 (0.91–1.07)	1.08 (0.99–1.18)		0.132
Never used	0.57 (0.53–0.61)^‡^	0.57 (0.53–0.62)^‡^	0.55 (0.50–0.62)^‡^		0.607
Thiazide diuretics					
Adherent	Reference	Reference	Reference		
Non‐adherent	1.07 (0.88–1.29)	1.21 (0.93–1.58)	0.94 (0.71–1.23)		0.180
Unknown	1.04 (0.95–1.13)	1.01 (0.90–1.15)	1.07 (0.94–1.21)		0.567
Never used	1.05 (0.98–1.12)	1.08 (0.99–1.19)	1.01 (0.92–1.11)		0.319
MRAs					
Adherent	Reference	Reference	Reference		
Non‐adherent	1.20 (1.09–1.33)^‡^	1.16 (1.02–1.33)*	1.26 (1.08–1.46)^†^		0.455
Unknown	1.00 (0.93–1.08)	1.03 (0.93–1.13)	0.97 (0.86–1.09)		0.432
Never used	0.77 (0.73–0.81)^‡^	0.77 (0.72–0.82)^‡^	0.76 (0.71–0.82)^‡^		0.793
Amiodarone					
Adherent	Reference	Reference	Reference		
Non‐adherent	1.06 (0.83–1.35)	0.99 (0.73–1.35)	1.18 (0.81–1.71)		0.479
Unknown	0.78 (0.68–0.90)^‡^	0.80 (0.67–0.95)*	0.75 (0.59–0.96)*		0.663
Never used	0.75 (0.69–0.81)^‡^	0.76 (0.69–0.84)^‡^	0.72 (0.62–0.82)^‡^		0.495
Digoxin					
Adherent	Reference	Reference	Reference		
Non‐adherent	1.00 (0.81–1.24)	0.96 (0.71–1.28)	1.06 (0.76–1.48)		0.640
Unknown	0.91 (0.82–1.01)	0.87 (0.75–0.998)*	0.97 (0.83–1.13)		0.290
Never used	0.89 (0.84–0.94)^‡^	0.89 (0.83–0.96)^†^	0.89 (0.81–0.96)^†^		0.843
Nitrates (isosorbide)					
Adherent	Reference	Reference	Reference		
Non‐adherent	1.08 (0.89–1.32)	0.98 (0.76–1.27)	1.25 (0.94–1.68)		0.215
Unknown	0.88 (0.81–0.95)^†^	0.85 (0.76–0.94)^†^	0.93 (0.81–1.06)		0.283
Never used	0.80 (0.75–0.85)^‡^	0.81 (0.75–0.88)^‡^	0.79 (0.72–0.87)^‡^		0.619

ACE, angiotensin‐converting enzyme; ARB, angiotensin receptor blocker; CI, confidence interval; COPD, chronic obstructive pulmonary disease; HR, hazard ratio; MRAs, mineralocorticoid receptor antagonists.

Significant interaction *P*‐values are in bold.

^a^ Duration in years since last chronic heart failure outpatient clinic visit or admission in period between 2012 and 2014.

* Significant at *P* ≤ 0.05.

^†^ Significant at *P* ≤ 0.01.

^‡^ Significant at *P* ≤ 0.001.

The relationship between the co‐morbidities and the PE was sex dependent. The sum of the regression coefficients of the co‐morbidities in the multivariable model in women (1.67) was larger than in men (0.65). In particular, RI, COPD/asthma, and DM1/2 had a stronger relationship with the PE in women. We found no significant difference between men and women in the prognostic value of medication (non‐)adherence.

## Discussion

### Main findings

In this analysis, based on a HIC database of >25 000 patients with CHF, we have shown that overall men demonstrated a worse prognosis compared with women. A broad range of co‐morbidities was significantly associated with increased risk of all‐cause mortality or HF admission. These associations were somewhat stronger in women than in men, in particular for RI, COPD/asthma, and diabetes, despite a higher prevalence in men. Men used more disease‐modifying drugs; however, adherence was similar between sexes. Non‐adherence to disease‐modifying drugs was related with a higher incidence of the PE, which again was similar between sexes.

Several large, national population‐based studies or registries on HF in Europe with >10 000 (10 190–88 195) patients have been published.^10^, ^11^, ^12^, ^13^, ^14^ To our knowledge, in the Netherlands, only two clinical registries on CHF exist, both having assessed European Society of Cardiology guideline adherence to HF medication.^12^, ^15^ The average age of patients across these large population‐based databases was 72–78 years, which is similar to our study (74 years). The percentage of women was 40–55% compared with 44% in our study, which is considerably higher than RCTs on HF with a median of about 29%.^16^ Considering the gender gap of ~25% between RCTs and the population at large reported for the US population,^16^ one would expect close to 50% female representation in a registry or insurance dataset. In the CHAMP‐HF registry, the average age (66 vs. 74 years) and percentage of women (29% vs. 44%) were lower compared with our study. This difference can be explained by the inclusion of solely HF with reduced ejection fraction (HFrEF) outpatients in CHAMP‐HF. An analysis of the Get With The Guidelines‐Heart Failure (GWTG‐HF) registry^17^ of 79 291 patients reported an average of 74–75 years and ~50% were women, which is relatively comparable with our study.

### Co‐morbidities

In this discussion, we will focus solely on the most relevant co‐morbidities with the highest impact on outcomes in overall and gender survival analysis. Studies on co‐morbidities have shown that most patients with CHF have >2 or even >5 co‐morbidities.^18^ Most patients in this dataset had ≥3 co‐morbidities, which is in line with the literature.

Across the previously described population‐based studies, prevalence of RI was 15–58%, COPD/asthma 15–32%, DM 25–43%, cerebrovascular disease 13–18%, and malignancy 5–21%.^10^, ^11^, ^13^, ^14^, ^17^, ^19^ In our study, this was 12%, 17%, 25%, 8%, and 25%, respectively. The higher rate for RI, COPD/asthma, and cerebrovascular disease in other studies is most likely inherent to the inclusion of mainly patients from outpatient clinics or after hospital discharge and a difference in classification method introducing some selection. In a cross‐sectional study of 122 630 CHF patients >65 of age, non‐cardiac co‐morbidities that had the highest risk for hospitalisations and overall mortality were COPD/bronchiectasis, renal failure, diabetes, depression, and other lower respiratory diseases.^20^ We showed that COPD, renal failure, and diabetes indeed have a high impact on these outcomes; however, depression was not significantly associated in this study. Chronic kidney disease (CKD) is associated with worse prognosis in patients with CHF.^21^ Analysis of the SwedeHF registry also showed a strong association of CKD with increased HF hospitalisation and all‐cause mortality.^14^ Interestingly, despite an extensive coverage of all cerebrovascular diseases using DRG codes and in sharp contrast to the registries where usually only stroke is examined, in this analysis, the prevalence was lower due to less selection. Malignancy (covering all neoplasms), however, was higher in our study as expected. Malignancy and cardiovascular disease have shared risk factors and therefore frequently coincide.^22^ Recently, Meijers *et al*. also demonstrated that HF can be considered a risk factor for incident cancer.^23^

In contrast to the overall mortality, no significant difference in mortality was observed between men and women who had been admitted for HF during follow‐up. We believe this discrepancy is due to sub‐selection of patients. Older women with HF with preserved ejection fraction (HFpEF) might be overrepresented closing the gap between the two groups. Sex differences exist in characteristics, aetiology, co‐morbidities, and prognosis. These differences might be attributed to the HF aetiology, left ventricular ejection fraction (HFrEF vs. HFpEF), and New York Health Association classifications, which were not available in this dataset. Women have a higher incidence of HF at older age, have more HFpEF, and suffer more from obesity, diabetes, and hypertension, while man have a higher incidence at a younger age with more HFrEF due to ischaemic aetiology.^24^ Furthermore, women generally live longer than men. In our study, women with RI, COPD/asthma, and diabetes had a worse prognosis, even though prevalence of RI was not significantly different, and COPD/asthma and diabetes were more common among men. In contrast, analysis of the SwedeHF registry showed that women were more likely to have CKD, with no sex difference on outcomes after adjustment.^14^ COPD is more common in men than women and is in line with the work of Lawson *et al*., who showed a 15% higher risk of mortality in women than men.^25^ Possible explanations are higher female age, pathophysiology, or delay in or poor response to treatment. Diabetes was more common among men in the SwedeHF registry, similar to ours.^14^ However, the study of Marra *et al*. demonstrated that women had more diabetes than men.^24^ Similar to our study, Johansson *et al*. showed that diabetes was a stronger predictor for mortality in women than men.^26^ This might be due to less evidence‐based management in women leading to a poor control of glucose levels.

### Medication use and adherence

Across the large population‐based studies, ACE‐I/ARBs use was 51–91%, beta‐blockers 52–90%, MRAs 12–56%, and loop diuretics 53–81%.^12^, ^13^, ^14^, ^15^, ^17^, ^19^ In our study, this was 85%, 83%, 40%, and 83%, respectively. Notably, there is a considerably large variation across studies, which might be related to differences in definition. Use of most of the aforementioned medications was particularly lower in the US registries, most likely due to lower guideline adherence.^17^, ^19^ Prescription rates in our study were comparable with the contemporary Dutch CHECK‐HF registry, a more clearly defined HF population, although we found lower rates for MRAs (40% vs. 56%) and loop diuretics (71% vs. 81%), which can be attributed to focus on HFrEF patients who are possibly more symptomatic (26%, New York Health Association III).^15^

As expected, ACE‐I/ARBs and MRAs had a significant association with the outcome in this analysis. However, beta‐blockers were not significantly associated, which is contrary to what would have been expected based on prior knowledge.^8^ We do not have a clear explanation for this phenomenon and hypothesize that it could be related to categorization of MPR, which leads to a loss of data and power. Moreover, women had a lower prescription rate of disease‐modifying drugs. However, adherence to medication and impact of adherence on outcome was not different between sexes. This could be in line with the work of Santema *et al*. who showed that women with HFrEF need lower doses (50% of recommended dose) of ACE‐I/ARB and beta‐blockers than men.^27^ This emphasizes the need for a sufficiently powered prospective cohort study for sex‐stratified analysis on use, dosage, and adherence of common HF medication on outcomes, also distinguishing for left ventricular ejection fraction.

### Strengths and limitations

Our study has several strengths. First, by using big data, a more representative, epidemiological overview compared with RCTs was given due to lower selection bias and a higher percentage of women. The results are more generalizable and apply to the CHF population at large in the Netherlands and possibly the rest of the EU. Second, extensive pharmacy data were available, and utilizing MPR with a cut‐off of 0.80^9^ and prescribed daily dose, because daily defined dose is a poor estimator,^28^ we reliably estimated medication adherence. This is the first step towards mapping patient compliance to medication. Last but not least, we compared our findings to multiple, large national registries, like the CHECK‐HF, to assess the validity of our findings. However, several limitations of this study should also be acknowledged using the checklist for retrospective database studies as a reference.^29^

First of all, limitations specific to study design are that data wrangling is more difficult due to complex data with an incomplete view. The lack of detailed medical data, such as HF aetiology, left ventricular ejection fraction, New York Health Association class, smoking status, body mass index, and laboratory values, complicates inferences on the results.^29^ Second, despite extensive adjustment for confounders in multivariable analysis, other (clinically) relevant confounders are lacking and residual confounding may be an issue. Regarding co‐morbidity selection, using the DRG/FKG method, a reasonable overview can be given. However, in most cases, the number of patients is susceptible to variation in criteria for diagnosis, and FKG data are prone to bias ‘healthier patients’. In our study, hypertensive disease, hypercholesterolaemia, and ischaemic heart disease showed to be protective of the outcome due to this reason. Therefore, caution is advised when interpreting these results. Third, due to the nature of data collection on insurance claims and the inherent delay that comes with it, not all data on hospitalisations might be present, leading to an incorrect representation. Furthermore, in HIC databases, coding can be subject to incorrect labelling (dyspnoea could be listed under CHF or COPD code), which can affect the validity of the results. This is however estimated at a maximum of 5%. Changes over time in codes can also lead to unreliable data,^29^ but quality check did not reveal any relevant changes that could affect the study findings.

## Conclusions

In a large, representative sample of the Dutch population, as captured in a HIC database, men with CHF had a 25% higher incidence of death or HF admission than women. The influence of co‐morbidities on the studied outcome was higher in women than in men, in particular for RI, COPD/asthma, and diabetes. No difference in sex for medication adherence and adherence in relation to the outcome was observed. These results underscore the merit of HIC databases as an addition to RCT data and demonstrate that additional research into sex differences in HF is warranted. To this end, the use and value of HIC databases should be further evaluated. Furthermore, treatment of HF should move towards a more patient‐tailored approach by sub‐classifying patients based on co‐morbidities and setting specific goals for these conditions that could potentially complicate HF treatment. More research is needed to determine these goals in (older) HF patients with multi‐morbidity and polypharmacy.

## Disclaimer

The views presented here are those of the authors. The European Commission is not responsible for any use that may be made of the information it contains.

## Conflict of interest

J.J.B. reports grants from Abbott, outside of the submitted work. All other authors have nothing to disclose.

## Funding

This project was supported by the European Union's Horizon 2020 research and innovation programme (780495).

## Supporting information


**Data S1**. Supplementary Appendix.
