# Supplementary material for: Impact of sex differences in co‐morbidities and medication adherence on outcome in 25 776 heart failure patients
Source: ESC Heart Fail. 2020 Nov 28;8(1):63–73. doi: 10.1002/ehf2.13113 (PMC7835621; doi:10.1002/ehf2.13113)
Supplement: Supplementary file 1 — Data S1. Supplementary Appendix. [file EHF2-8-63-s001.docx]

Supplementary Appendix

This appendix has been provided by the authors to give readers additional information about their work.

Supplement to:

**Impact of sex differences in comorbidities and medication adherence on outcome in 25,776 heart failure patients**

Muhammed T. Gürgöze^1^, Onno P. van der Galiën^2^, Marlou A.M. Limpens^3^, Stefan Roest^1^, René C. Hoekstra^2^, Arne S. IJpma^4^, Jasper J. Brugts^1^, Olivier C. Manintveld^1^, Eric Boersma^1^

^1^Departement of Cardiology, Thorax Center, Erasmus MC, University Medical Center Rotterdam, Rotterdam, The Netherlands

^2^Zilveren Kruis Achmea, Leusden, The Netherlands

^3^Departement of Epidemiology, Erasmus MC, University Medical Center Rotterdam, Rotterdam, The Netherlands

^4^Departement of Pathology, Erasmus MC, University Medical Center Rotterdam, Rotterdam, The Netherlands

**Supplementary Methods**

**Selection of CHF patients**

In the period of January 1^st^ 2012 until December 31^st^ 2014 patients with chronic heart failure (CHF) were identified using the following Diagnose Behandeling Combinatie (DBC) codes:

099899045

099899046

099899066

099899067

099899068

Additionally, patients had to have used at least one medication within the cardiovascular system, classified as C based on World Health Organization Anatomical Therapeutic Chemical Classification index and Defined Daily Dose (WHO ATC/DDD) in the same period.

If one of the above CHF DBC codes was registered at least once AND at least one of the medication in class C of WHO ATC/DDD in the given time period, the patient was considered a CHF patient.

**Definition demographics & socio-economic status**

Baseline age was defined as the age at January 1, 2015. Sex and marital state of patients was retrieved from the database. Socio-economic status was determined using four number zip code and status scores of 2014 supplied online by Netherlands Institute for Social Research (SCP). These scores are a composite measure of an inhabitant’s characteristics for that neighborhood: education, income and occupation. A low score (can be negative) equals a low socio-economic status. Income level was determined separately using SCP data based on the combination of full zip code published in 2015 over the year 2014. In this publication the median of the standardized income per household was compared with the distribution of incomes for all households in the Netherlands. The income was categorized in levels ranging from 0 (< 15,900€) to 10 (> 34,600€).

**Selection of Comorbidities**

Clinically relevant comorbidities were selected using either Diagnosis-related group (DRG), which include 141 groups in the database, or Pharmacy-based cost group (FKG), covering considerably less diagnoses (24). If available both DRG and FKG were used to ensure full ascertainment of comorbidity information. Below is a list of the classification used to retrieve comorbidity information per comorbidity:

1. Arrhythmia DRG [I46-I49]
2. Cerebrovascular disease DRG [I60-I69]
3. COPD/Asthma DRG [J44] + FKG
4. Depression FKG
5. Diabetes Mellitus 1/2 DRG [E10-E13] + FKG
6. Hypercholesterolemia FKG
7. Hypertensive disease DRG [I10-I15]
8. Ischemic heart disease DRG [I20-I25] (includes ischaemia/PCI/CABG)
9. Malignancy DRG [all neoplasms] + FKG
10. Renal insufficiency DRG [N17-N19]
11. Thyroid dysfunction DRG [E05-E07] + FKG
12. Valve disease DRG [I120]

**Selection of ATC medication**

Selection of heart failure medication and other medication was done using the World Health Organization Anatomical Therapeutic Chemical Classification index and Defined Daily Dose (WHO ATC/DDD). Usage of combination medications was assigned to each group included in the combination.

**Definition of medication adherence**

The obtained refill rates were categorized into four groups:

1. Adherent; used medication in ATC group and MPR ≥ 0.80
2. Non-adherent; used medication in ATC group and MPR <0.80
3. Unknown; patient used medication, but:
   1. Medication was supplied two or less times
   2. Prescribed daily dose was not reliable
4. Never used; patient has never used this medication in 2012-2014

**Selection of hospital admissions**

Acute or chronic heart failure related hospital admissions during the follow-up period from January 2015 until April, 2018 were determined using the following DBC codes:

099899049

099899024

099899050

099899108

099899045

099899067

099899046

099899068

099899066

Detailed information of health activities linked with the DBC codes were available from 2015 onwards, including nursing days. These were used to determine hospitalisation and length-of-stay.
